# Blue light influences negative thoughts of self

**DOI:** 10.1093/sleep/zsaf034

**Published:** 2025-02-25

**Authors:** Malisa T Burge, Ronel A Lumapas, Alicia C Lander, Brianna G Thomas, Andrew J K Phillips, Sean W Cain

**Affiliations:** School of Psychological Sciences and Turner Institute for Brain and Mental Health, Monash University, Melbourne, VIC, Australia; School of Psychological Sciences and Turner Institute for Brain and Mental Health, Monash University, Melbourne, VIC, Australia; School of Psychological Sciences and Turner Institute for Brain and Mental Health, Monash University, Melbourne, VIC, Australia; Flinders Health and Medical Research Institute (Sleep Health), Flinders University, Bedford Park, SA, Australia; School of Psychological Sciences and Turner Institute for Brain and Mental Health, Monash University, Melbourne, VIC, Australia; Flinders Health and Medical Research Institute (Sleep Health), Flinders University, Bedford Park, SA, Australia; Flinders Health and Medical Research Institute (Sleep Health), Flinders University, Bedford Park, SA, Australia

**Keywords:** circadian, self-referencing, blue-light, depression, cognition, HDDM

## Abstract

Darkness is equated with sadness. This study explored how light that differentially impacts non-visual photoreception (blue-enriched vs. blue-depleted light) affects how we feel about ourselves. In a repeated-measured design, 35 participants (22 female participants, 13 male participants, *M*_age_ = 20.29, *SD* = 2.09) completed the self-referential encoding task (SRET) under both blue-enriched or blue-depleted light conditions, with light conditions randomized and counterbalanced between sessions. The SRET involved participants deciding whether positive (e.g. “good”) and negative (e.g. “terrible”) words were self-descriptive. Trial-by-trial performance analysis using logistic mixed effects models revealed that blue-enriched light significantly increased the likelihood of rejecting negative words as self-descriptive. A hierarchical drift-diffusion model (HDDM) further examined latent decision-making processes and found evidence accumulation to be faster under blue-enriched light when rejecting negative descriptors, suggesting rejecting negative self-descriptors was easier under blue-enriched light. We find light can acutely influence self-perception, with blue-enriched light decreasing negative self-thoughts.

## Graphical Abstract

**Graphical Abstract:**
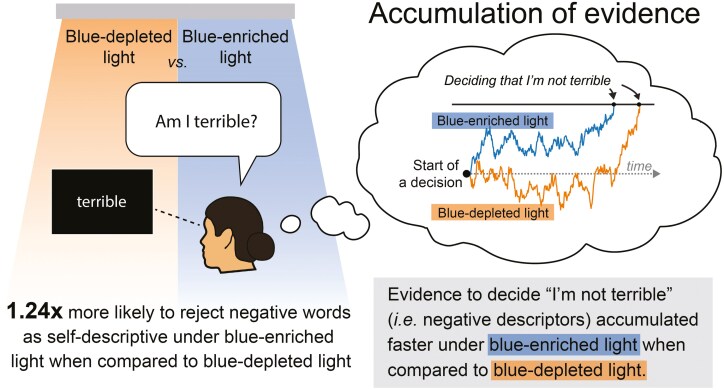

**Significance Statement**

In industrialized countries, there is ubiquitous availability of brighter, blue-enriched LED lights, including home lights, televisions, and handheld devices. There is also pervasive light pollution in our outdoor areas. Our work reveals a powerful motivation in humans toward this blue-enriched light: reducing self-directed negative thoughts. In humans, non-visual photoreception quiets areas of the brain involved in negative emotions and our work shows that simply changing the amount of *blue* in a light impacts how we feel about ourselves. As non-visual photoreception may be downregulated in depression, this may also be key to understanding neural mechanisms of depressed thought and provides a mechanistic understanding of light interventions to improve mood.

In the opening lines of Emily Dickinson’s poem, “We grow accustomed to the Dark/When Light is put away” we find a reflection of a fundamental human experience—that the presence of darkness is associated with feelings of sadness and emptiness. However, an association of darkness with sorrow is more than a metaphor. An emerging body of research indicates that this insight may have a biological basis. In humans, light improves mood [1]. Conversely, dimmer daytime light is associated with mood disturbances such as increased risk of depression and self-harm behavior [2]. Additionally, depressive symptoms in seasonal affective disorder (thought to have affected Dickinson herself) vary by photoperiod, with worse mood when there is less daylight [3, 4].

The direct effects of light on mood are mediated by a non-visual mechanism that can be independent of the circadian clock [1]. A group of retinal cells called intrinsically photosensitive retinal ganglion cells (ipRGCs) containing the photopigment melanopsin receive light information from our external world and preferentially respond to short-wavelength, “blue” light (~480 nm) [5]. This light information is projected via ipRGCs to non-visual brain regions involved in mood and cognition, acutely changing mood [6]. In animal models, retinal illumination has been shown to decrease the activity of the lateral habenula [7], an area in the brain involved in motivation, sensitivity to reward, and emotional processing [8]. In humans changes in light alter the activity of the lateral habenula [9]. Furthermore, the perihabenula (adjacent to the lateral habenula) receives direct input from ipRGC cells, which has been shown to impact mood via downstream projections to frontal regions such as the ventromedial prefrontal cortex (vmPFC) [10]. These non-visual pathways are hypothesized to contribute to the light-mediated changes in mood of certain mood disorders [11].

Negative versus positive self-perceptions can be measured by the self-referential encoding task (SRET) [12, 13]. Through self-referential processing—the process by which we relate information to ourselves individuals make decisions about whether positive (e.g. joyful, gentle, and funny) and negative (e.g. disloyal, alone, and dumb) words describe themselves [14]. SRET metrics, such as endorsement of negative words and rejection of positive words as self-descriptive, have been strongly associated with depression severity [13, 15]. Additionally, reaction time (RT) and choice data from the SRET can be used to model latent processes of decision-making with the drift-diffusion model (DDM) [16]. In this model, decisions are assumed to be made by a noisy process that accumulates information over time, from a starting point *z* toward one of two response criteria or boundaries: *a* and 0 [16]. When a boundary is reached, a response is initiated. The speed at which evidence accumulates, termed the drift rate (*v*), reflects the preference for one option over another, where a faster drift rate signifies ease in making a particular choice, resulting in more consistent decisions [17] (see Figure 1 for example drift-diffusion process). In the SRET, a faster drift rate reflects the relative ease in rejecting or endorsing an adjective [13], leading to quicker and more consistent decisions [18]. Conversely, a varying bias *z* on the SRET reflects an a priori bias towards endorsement or rejection of self-descriptors before evidence begins to accumulate [19].

**Figure 1.**
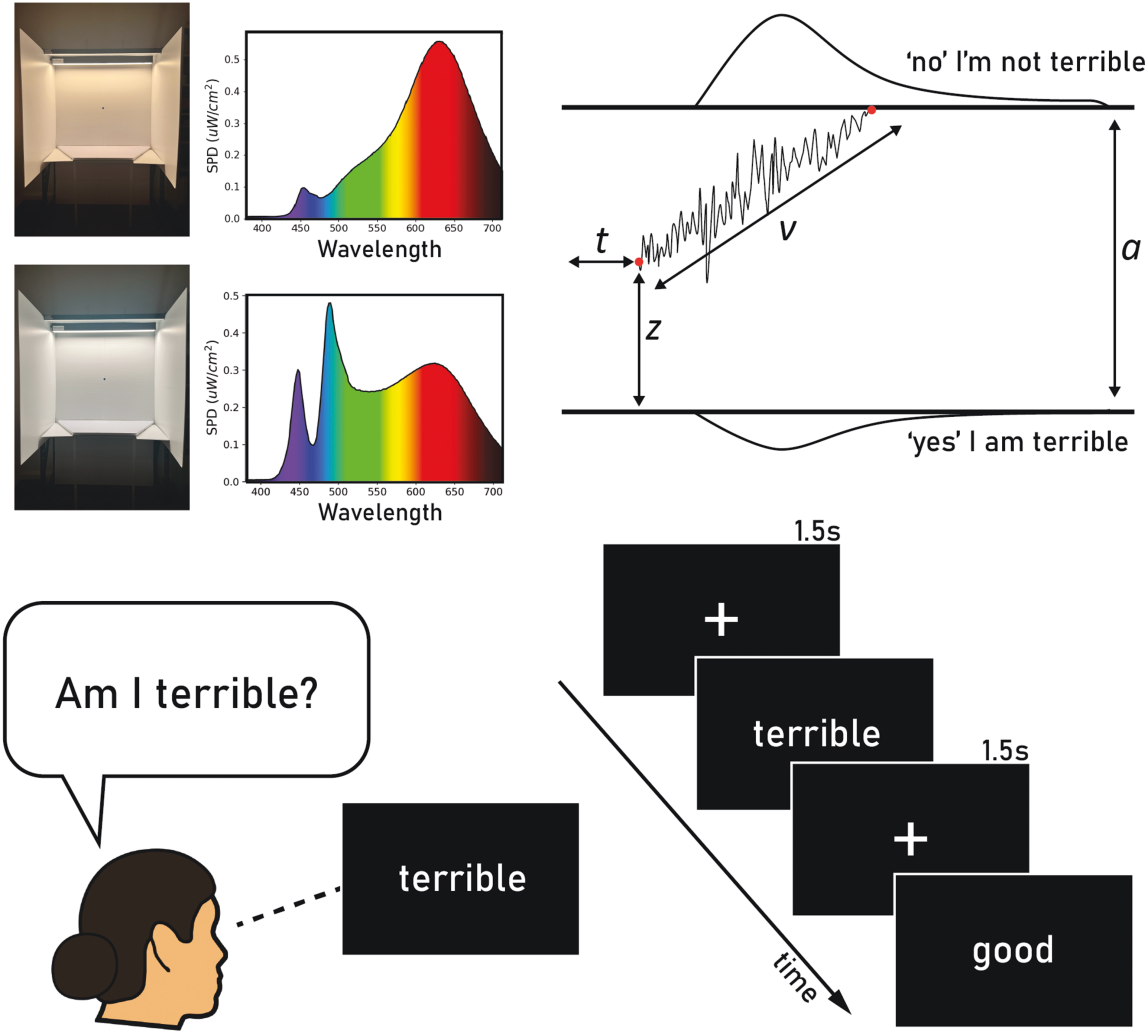
Top left: spectral qualities and visual differences of blue-enriched (bottom) and blue-depleted (top) light conditions. Top right: example of the drift-diffusion process in response to “terrible.” Key parameters include *a* – boundary separation, indicating the amount of information required before a decision is made, *v* – drift rate, reflecting the average speed at which evidence is accumulated for one decision over the other, *t* – non-decision time, the time taken for processes other than decision-making like response execution, and *z* – bias parameter, an a priori bias to “yes” or “no.” The drift rate here is shown moving towards the “‘no’ I’m not terrible” decision boundary. Bottom left: shows a participant deciding whether they are “terrible” or not in the Self-Referential Encoding Task. Bottom right: a visual representation of two trials in the Self-Referential Encoding Task.

Many of the downstream target sites of ipRGCs relate to self-referential processing. For example, vmPFC activity is associated with increased self-focused thinking [20]. Given the non-visual pathways by which light acts, we hypothesized that blue-enriched light preferentially activating ipRGCs may change how we perceive ourselves. In this study, we investigated how blue light affects thoughts of self by using light of the same visual brightness but differing effects on the activation of melanopsin. Given the effect of light on mood in humans, we hypothesized that melanopsin-activating blue light would reduce negative self-evaluation on the SRET (i.e. rejecting more negative words and endorsing more positive words as self-describing). Additionally, as choices are dictated by latent processes of decision-making, such as the rate at which we accumulate evidence and an a priori bias towards certain types of responding, we also expected blue light to influence the parameters of the DDM [16]. Here, we provide evidence that blue light reduces negative self-evaluation during the SRET. Additionally, we explain the mechanism by which blue light affects responses using the DDM. Our findings suggest that blue light can acutely improve self-perceptions.

## Methods

Thirty-five healthy individuals aged between 18 and 27 (22 female participants, 13 male participants, *M*_age_ = 20.29, *SD* = 2.09) were recruited from the general community and a pool of past participants consented to future contact. Utilizing a within-participants, repeated-measures design, the study assessed how light impacting non-visual photoreception influences thoughts of self. Participants underwent two sessions, 2 weeks apart, completing the SRET under two lighting conditions: blue-depleted (peak wavelength = 631 nm) and blue-enriched (peak wavelength = 490 nm) which were randomly allocated and counterbalanced between sessions. Participant demographics are provided in Table 1.

**Table 1.** The Demographic of Participants by Participant Sex

	Total	Female participants	Male participants
*N*	35	22	13
Age *M*(*SD*)	20.23(2.13)	19.91(1.77)	20.77(2.62)
BMI *M*(*SD*)	22.84(3.13)	23.10(3.39)	22.48(2.71)
Ethnicity			
South-East Asian (%)	42.86%		
South Asian (%)	22.86%		
North-East Asian (%)	14.29%		
Caucasian (%)	14.29%		
Did not specify (%)	5.71%		

Eligible participants were identified via a screening questionnaire and provided consent online or in person. The study was approved by the Monash University Human Research Ethics Committee (MUHREC), approval number 32054. Prior to each session, participants were asked to obtain a normal night of sleep and to abstain from alcohol and caffeine for at least 24 hours. Laboratory visits were scheduled from approximately 4–7 hours after participants’ usual wake time, with both sessions occurring at roughly the same time of day at least two weeks apart.

Upon arrival, participants sat under the light structure (eye level 123 cm from the floor and 63.3 cm from a fixation point; see Figure 1), starting with an initial “dim” period (<3 lux) for five minutes. Participants then completed the rest of the session under the blue-enriched (~490 nm) or blue-depleted (~631 nm) light conditions, which aimed to differentially impact non-visual photoreception. After the lights were turned on, participants were instructed to direct their gaze to a fixation point for 5 minutes to standardize the light exposure. In total, each session lasted up to 90 minutes. Administration of the SRET occurred amongst a battery of other cognitive tasks in the broader study during light exposure. Participants completed the SRET at approximately 12 minutes after their light exposure began.

Trials of the SRET involved deciding whether positively (e.g. good) or negatively (e.g. terrible) valanced adjectives were self-descriptive by indicating “yes” or “no” on a keyboard, see Figure 1 for visualization of the task. Participants were asked to complete trials as quickly and as accurately as possible. RT (speed of choice) and choice data (endorsement or rejection of valanced adjectives) on the SRET were used to model decision-making. Two participants were not included in the final analysis due to falling asleep during the sessions. At the end of the final session, participants were asked to what extent they were aware of differences in the lights between sessions and provided written qualitative descriptions of differences between lights between the sessions. Only 26% of the participants (9 out of 35) were able to correctly identify differences in hue between sessions.

## Materials

### Screening questionnaire

Potential participants completed questions about demographic information online (e.g. age, sex, gender, height, weight, etc). Eligible participants did not have regular medication use, any current medical conditions, or personal mental health illness, and female participants were not pregnant or breastfeeding.

### Melagen lighting device

Light exposure was controlled using the Melagen lighting device (Versalux Lighting Systems, Mitchem, Victoria). This is an LED light source (CRI89), with two light stimuli having a correlated color temperature of either ~2200K (λp = 631 nm) or ~4200K (λp = 490 nm). Both light stimuli were delivered at similar photopic lux (photopic illuminance) and similar irradiance of 198.64 lux and 73.08 μW/cm2 for the blue-enriched condition and 201.49 lux and 78.90 μW/cm2 for the blue-depleted light condition (intensity was assessed using ColourMunki, X-Rite, USA; spectral characteristics assessed using a MK350N Spectrometer, UPRTek, Taiwan). Melanopic equivalent daylight illuminance (EDI) was 173.25 lux and 70.26 lux for the blue-enriched and blue-depleted conditions, respectively. Melanopic EDI quantifies melanopsin stimulation and was calculated using the α-opic, Toolbox which determines effective illuminance for different photoreceptor types based on the spectral power distribution of a light source [21]. Participants’ eye height and distance from the backboard were standardized to ensure equal exposure across participants. Before the lighting was turned on, participants completed a “dim” adaption period of <3 lux for five minutes. During this time, participants were instructed to remain still and focus on a fixation point. After five minutes the lighting device was turned on and participants were instructed to look at the fixation point for a further five minutes to standardize exposure. After five minutes of light, exposure participants began the battery of cognitive tasks.

### Self-referential encoding task

An adaption of Derry and Kuiper’s (1981) SRET was administered on a computer monitor [14] using PsychoPy 3 [22]. The screen was covered by a neutral density filter to minimize light exposure from the computer screen. The task required individuals to decide whether a word could be used to describe themselves with the binary option of “yes” or “no.” Participants indicated their response by pressing the “P” or “Q” key with their respective index fingers on a keyboard, for “yes” and “no,” respectively. Each trial ended only after the participant had made a response. The end of a trial was proceeded by a 1500 ms intertrial interval (ITI) which included a white cross in the middle of the screen which participants were instructed to “look at.” In total, the SRET consisted of three trial blocks, with 52 adjectives (26 positive, 26 negative; 156 words in total) being displayed one at a time in random order. The number of trials included in this study was previously suggested by Dainer-Best et al. (2018) who used this task in conjunction with DDM [13]. Positive and negative words used in the SRET were consistent with Dainer-Best et al. [13]. For a subset of participants, “silly” was replaced with “dumb” consistent with Dainer-Best et al. [13].

### Light perception questions

At the conclusion of the second session, participants were asked to describe differences in the lights between sessions. Accurate identification of differences between sessions was determined based on qualitative descriptions. Specifically, if the participants identified differences in hue for the respective sessions, for example, the blue-enriched session was “whiter” or “bluer,” and the blue-depleted session was “warmer,” “yellow,” or “amber.”

## Statistical Methods

### Simple summary statistics

To analyze simple summary statistics of performance on the SRET, we calculated the proportion of positive words endorsed and negative words rejected for each participant in the blue-enriched and blue-depleted conditions.

To compare differences between the two light conditions, we conducted two paired-sample *t*-tests comparing proportion of positive words endorsed by light condition, and comparing proportion of negative words rejected by the light condition. These tests were one-tailed, with the expected direction assuming a larger proportion of positive words endorsed in the blue-enriched condition and a larger proportion of negative words rejected in the blue-enriched condition. Normality was assessed by visual inspection of the difference in proportion between the blue-enriched and blue-depleted light conditions.

### Trial-by-trial performance

Logistic mixed-effects models were fitted to investigate the influence of blue-enriched or blue-depleted light on the likelihood of positive self-evaluation (i.e. rejecting negative or endorsing positive words as self-describing) separately for positive and negative words, analyzed on a trial-by-trial basis (estimated using ML and BOBYQA optimizer). Random intercepts for each individual were included to control for individual differences across trials and sessions (*formula: ~1 | participant*). Fixed effects included “condition” (blue-enriched or blue-depleted light), “reaction time” (in seconds), “sex” (male or female participants), and “hue” (correct or incorrect identification of hue differences in sessions). We tested several model configurations including main effects models, interaction models, and three-way interaction models. These models allowed us to assess the main effects of variables of interest, as well as whether models were moderated by sex and hue awareness (see Supplementary For Model Comparison).

Given the risk of over-fitting due to multiple interaction, terms we assessed model fit using the Bayesian information criterion (BIC) corrected for small sample sizes, as it applies a larger penalty for more complex models. Additionally, for all models, standardized parameters were obtained by fitting the model on a standardized version of the dataset. To calculate 95% Confidence Intervals (CIs) and *p*-values, a Wald z-distribution approximation method was used. Model diagnostics were performed using simulated residuals to check assumptions of the model, including homoscedasticity and dispersion using the DHARMA package [23].

### Drift diffusion model

The DDM is a computational framework used to model the latent decision-making process in two alternative forced-choice decision-making (Figure 1) [16]. It assumes that the decision-making process is an accumulation of noisy evidence (drift rate; *v*) over time until a decision threshold is reached. Evidence is accumulated at a rate (*v*) from a starting point (*z*), until one of the two decision boundaries is reached, representing two possible choices, in this case endorsing or rejecting a word (e.g. “yes, I am terrible,” or “no, I am not terrible”). The model includes a term that accounts for non-decision processes, such as the time it takes to encode the stimulus and to execute a response termed non-decision time (*t*). The distance between the two possible decisions is represented by the boundary separation parameter (*a*), which reflects the amount of evidence required before a decision is executed. Unlike traditional accuracy tasks, responses on the SRET are considered neither incorrect nor correct. We instead coded responses as “0” = “yes, this word describes me” and “1” = “no, this word does not describe me.”

In the current study, we employed hierarchical DDMs (HDDM) to examine the latent processes involved in decision-making [24]. HDDM offers several advantages over DDM for our study design: (1) HDDM requires fewer data points per participant/condition than nonhierarchical methods; (2) it allows us to estimate condition-specific parameters for each light condition; (3) the model simultaneously estimates both group-level and individual-level parameters allowing within-individual estimation of condition effects (i.e. hierarchical). Additionally, the HDDM uses a Bayesian framework to enhance statistical power by utilizing established DDM findings to inform model estimates (prior informed posteriors) and constrain ranges of parameter estimates to plausible values [24]. Finally, statistical power is also enhanced by incorporating all trials allowing trial-by-trial variability. To assess significant differences between condition parameters, we examined the posterior distributions of the parameters. Specifically, we considered differences significant when less than 5% of the probability density mass of the posterior distributions overlapped between conditions.

Variants of a 2 × 2 within-participants HDDM regression model were run to compare changes in parameter posterior distributions across the two factors: light condition (blue-enriched or blue-depleted light), and stimulus type (positive or negative words). The general form of our regression model was:


$$\begin{array}{l} parameter\;∼\;0+C(condition):C(stimulus) \end{array}$$

Where “condition” represents the light condition, “stimulus” represents positive or negative words on the SRET, and ‘parameter’ indicates the DDM parameter being estimated (*v* for drift rate, *z* for starting point bias, or *t* for non-decision time). The ‘0’ indicates a model without an intercept term, allowing for the estimation of each condition:stimulus combination. Six model variants were tested, systematically varying the following parameters: *t*, *v*, *vt*, *vz*, *z*, *z,t*. For each variant, the specified parameters were allowed to vary by condition and stimulus type according to the model formula, while other parameters were held constant across conditions. Models allowing boundary separation (*a*) to vary were attempted but these models consistently failed to converge. The persistent failure to fit models with varying boundary separation may indicate that changes in this parameter are unlikely to account for choice and RT distributions. We utilized the Markov chain Monte Carlo (MCMC) algorithm to estimate parameter distributions for each model. For each model 30 000 samples were drawn, the first 5000 samples were ‘burned’ to reduce the effect of early estimations on model accuracy as these samples had less convergence, and only every 10th sample was retained to reduce autocorrelation. The six model variants successfully converged, as verified by trace plots of MCMC chains. Rapid decay in all autocorrelation plots indicated minimal autocorrelation within chains (Supplementary for plots). As more complex models better predict data, models can become infinitely more complex. To balance the complexity of models by fit of the model, a Deviance Information Criterion (DIC) penalized model complexity for each increase in complexity—degrees of freedom and was to identify the best model.

## Results

### Descriptive statistics and simple summary statistics

Descriptive statistics for SRET performance under blue-enriched and blue-depleted light conditions are presented in Table 2. This table summarizes reaction time (RT) and the proportion of response type for both negative and positive words across the two light conditions. For negative words, participants showed a tendency to reject rather than endorse words in both light conditions. Under blue-enriched light, 85.0% of negative words were rejected compared to 82.6% under blue-depleted light. However, a one-tailed paired sample *t*-test indicated that the difference (mean difference = 2.4%) in the proportion of rejecting negative words was not significantly different between conditions, *t*(32) = 1.40, *p* = .086. For positive stimuli, participants demonstrated a tendency to endorse rather than reject words. Under blue-enriched light, 72.4% of positive words were endorsed, while under blue-depleted light, this percentage was slightly higher at 73.3%. A one-tailed paired sample t-test indicated that the difference (mean difference = −0.01%) in the proportion of endorsing positive words was not significantly different between conditions, *t*(32) = −0.49, *p* = .686. For both tests, the difference in the proportion of responses of the blue-enriched and blue-depleted conditions was normally distributed.

**Table 2.** Reaction Times (RT) in Seconds and Percentages for the Self-referential Encoding Task Performance Under Blue-Enriched and Blue-Depleted Light Conditions

	Blue-enriched light condition			Blue-depleted light condition		
	*n*	%	RT *M*(*SD*)	*n*	%	RT *M*(*SD*)
Negative stimuli						
Endorsing negative word	387	15.0%	1.10(0.69)	448	17.4%	1.07(0.63)
Rejecting negative word	2187	85.0%	0.84(0.51)	2126	82.6%	0.86(0.39)
Positive stimuli						
Endorsing positive word	1864	72.4%	0.85(0.44)	1887	73.3%	0.82(0.35)
Rejecting positive word	710	27.6%	0.99(0.49)	687	26.7%	1.05(0.62)

**Figure 2.**
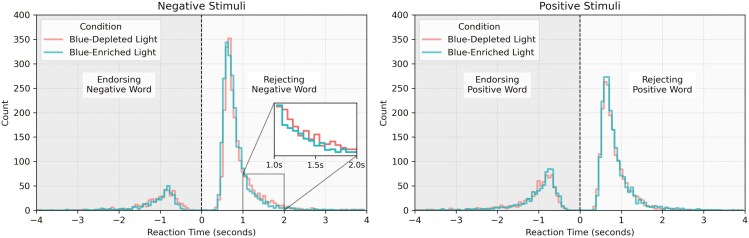
Distribution of reaction times in seconds for positive and negative stimuli on the self-referential encoding task under blue-depleted and blue-enriched light conditions. The left panel shows responses to negative stimuli, where the reaction time distribution between −4 to 0 (dark gray region) represents responses for endorsing a negative word as self-descriptive, and responses 0 to 4 (light gray region) represent responses for rejecting a negative word. The right panel shows responses to positive stimuli, where the reaction time distribution between −4 to 0 (dark gray region) represents responses for rejecting a positive word as self-descriptive, and responses 0 to 4 (light gray region) represent responses for endorsing a positive word. Inset plots are provided for rejecting negative words at 0.0–0.75 seconds and 1.0–2.0 seconds. The inset plot at 0.0–0.75 seconds shows a leading reaction time distribution for the blue-enriched condition. The inset plot at 1.0–2.0 seconds shows a trailing distribution for the blue-depleted condition.

RT distributions for negative and positive stimuli of SRET responses are plotted in Figure 2. Overall participants had higher counts of rejecting negative words and endorsing positive words as self-descriptive. The majority of responses were less than 1 second. For negative stimuli, visual inspection of the RT distributions showed a leading edge for the blue-enriched condition, and a trailing tail for the blue-depleted condition.

### Trial-by-trial performance

#### Positive words.

A set of logistic mixed effects models predicting odds of positive self-evaluation for positive words on the SRET was conducted. Positive self-evaluation (endorsing positive words as self-descriptive) was best predicted by the model with main effects of condition and RT, formula: *Positive self-evaluation ~ (1|participant) + condition + RT*. Comparison of models using BIC and model estimates are provided in the Supplementary Material document. Table 3 provides the results of the logistic mixed effects model.

**Table 3.** Logistic Mixed Effects Model Predicting Odds of Positive Self-evaluation for Positive Words by Light Condition and Reaction Time

	Odds of positive self-evaluation (Endorsing positive word)		
Predictors	Odds ratios	CI	*p*
(Intercept)	9.24	5.18 – 16.49	<.001
Condition (blue-depleted light = reference)	0.91	0.78 – 1.06	.222
Reaction time (seconds)	0.44	0.36 – 0.53	<.001

Blue-depleted light was the reference category. The odds ratio of condition indicates the change in odds for blue-enriched light when compared to blue-depleted light. CI = Confidence Interval; *p* = *p*-value. Random effects: Participant-specific intercept variance (σ²) = 3.29; intraclass correlation coefficient (ICC) = 0.43 (43% of variance due to between-participant differences); N participants = 33; Observations = 5148. Model fit: Marginal *R*² = 0.024 (variance explained by fixed effects), Conditional *R*² = 0.443 (variance explained by full model). Odds ratios represent the change in odds of endorsing a positive word as self-descriptive for a one-unit increase in the predictor.

The model exhibits a significant degree of overall explanatory power, as indicated by a conditional *R*^2^ value of 0.44. The proportion of variance explained by the fixed effects alone was 2.4% as indicated by marginal *R*^2^. Random effects revealed a considerable amount of individual variance in intercepts (σ^2^ = 3.29). The intraclass correlation coefficient (ICC) indicated that 43% of the total variance in endorsing or rejecting positive words was attributable to between-person differences (τ_00_ = 2.40). The light condition did not significantly predict the odds of positive self-evaluation to positive words (*p* = .222). RT did significantly predict the odds of negative self-evaluation (*p < *.001) as longer RTs reduced the odds of positive word endorsement. For every 1-second increase in reaction time, the odds of endorsing a positive word decreased by 56%.

### Negative words

A series of logistic mixed effects models were conducted to predict the odds of positive self-evaluation to negative words in the SRET. Positive self-evaluation (rejecting negative words as self-descriptive) was best predicted by the model with main effects of condition and RT, formula: *Positive self-evaluation ~ (1|participant) + condition + RT*. A comparison of models using BIC and model estimates are provided in the supplementary document. Table 4 provides the results of the logistic mixed effects model.

**Table 4.** Logistic Mixed Effects Model Predicting Odds of Positive Self-evaluation to Negative Words by the Light Condition and Reaction Time

	Odds of positive self-evaluation (rejecting negative word)		
Predictors	Odds ratios	CI	*p*
(Intercept)	12.07	7.06 – 20.63	<.001
Condition (blue-depleted light = reference)	1.24	1.05 – 1.47	.011
Reaction time (seconds)	0.68	0.58 – 0.80	<.001

Blue-depleted light was the reference category. The odds ratio of condition indicates the change in odds for blue-enriched light when compared to blue-depleted light. CI = Confidence Interval; *p* = *p*-value. Random effects: Participant-specific intercept variance (σ²) = 3.29; intraclass correlation coefficient (ICC) = 0.38 (38% of variance due to between-participant differences); N participants = 33; Observations = 5148. Model fit: Marginal *R*² = 0.009 (variance explained by fixed effects), Conditional *R*² = 0.390 (variance explained by full model). Odds ratios represent the change in odds of rejecting a negative word as self-descriptive for a one-unit increase in the predictor.

The model exhibits a significant degree of overall explanatory power, as indicated by a conditional *R*^2^ value of 0.39. The proportion of variance explained by the fixed effects alone was 1% as indicated by marginal *R*^2^. Random effects revealed a considerable amount of individual variance in intercepts (σ^2^ = 3.29). The ICC indicated that 38% of the total variance in endorsing or rejecting a negative word was attributable to between-person differences (τ_00_ = 1.88). The light condition did significantly predict the odds of positive self-evaluation (*p* = .011), with the blue-enriched condition increasing the odds of rejecting a negative word as self-descriptive by 24%. RT did significantly predict the odds of negative self-evaluation (*p* < .001) as longer RTs reduced the odds of negative word rejection. For every 1-second increase in reaction time, the odds of rejecting a negative word decreased by 32%.

### Latent decision-making processes

**Figure 3.**
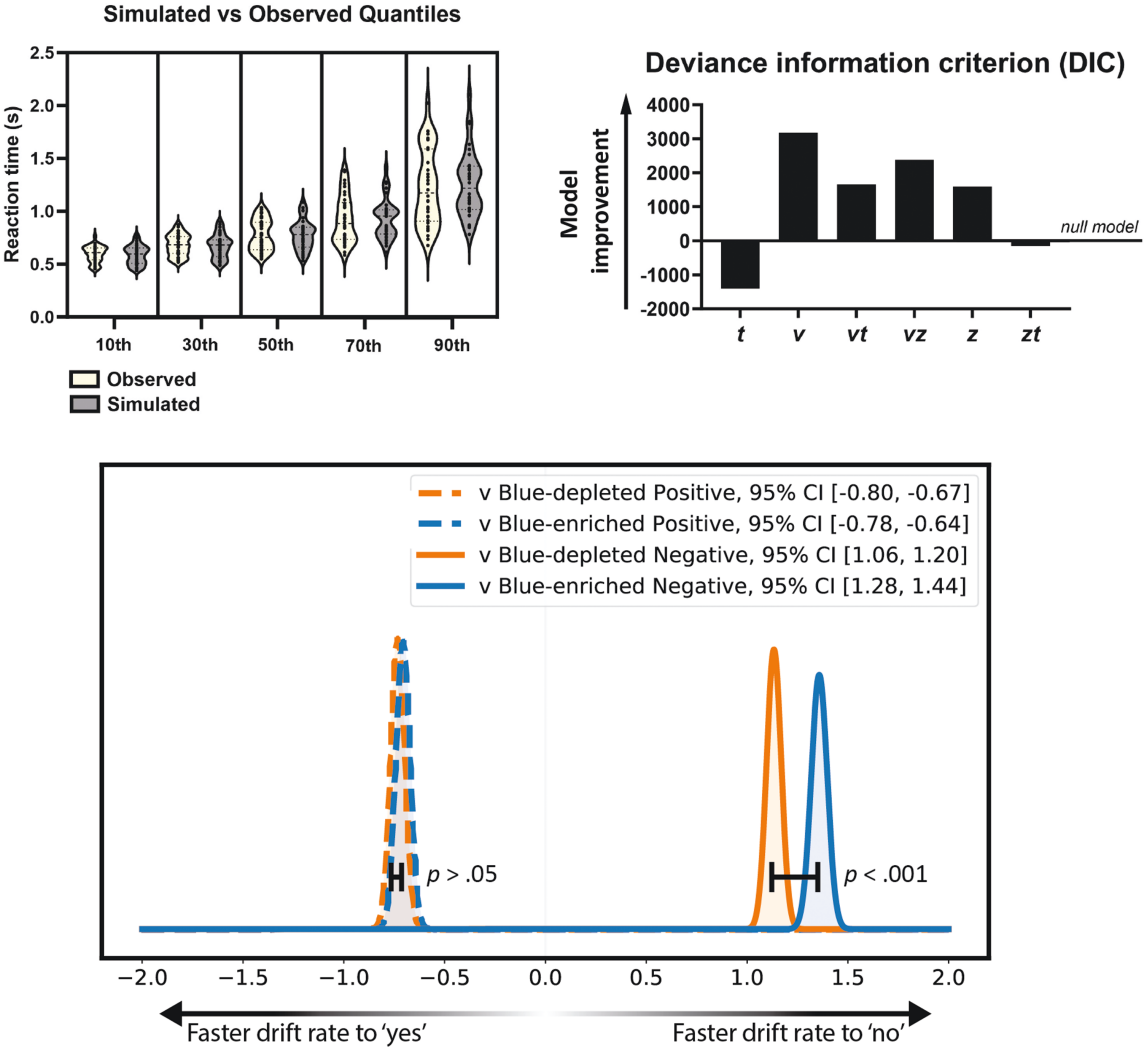
Model validation, comparison, and parameter distributions in response to different stimuli and light conditions. Top left: comparisons between observed reaction times (depicted in yellow on the left) and simulated reaction times (depicted in gray on the right) based on the best fit HDDM model (varying *v*), at the 10th, 30th, 50th, 70th, and 90th percentiles. Simulated percentiles were calculated using HDDM output of each participant’s parameter estimations of *a* (boundary separation), *t* (non-decision time), and *z* (bias), and group-level values of *v* (drift rate). Observed percentile values were adjusted by removing outliers that exceeded 3.29 standard deviations from the mean. Top right: DIC (Deviance Information Criterion) comparison of the best-fitting model is displayed relative to a model where no parameter varies—higher scores correspond to a better model fit. The model where only *v* (drift rate) varies is the best fit. Bottom: posterior distributions of *v* drift rate for positive and negative SRET (self-referential encoding task) stimuli under the blue-enriched and blue-depleted light conditions. Significant differences were observed in the drift rate for negative words (non-dashed lines) between the blue-enriched and blue-depleted light conditions, but no differences for positive words (dashed lines).

Amongst a set of candidate HDDM models, the best-fit model was one in which only the drift rate varied (see Figure 3 for DIC comparison of models). To assess the robustness of this model, we ran five models where only the drift rate varied and evaluated both between-chain and within-chain estimates of model parameters to ensure consistency across chains. R-hat values for every parameter estimate were <1.01, confirming convergence (Supplementary Material for chains).

The mean posterior distributions of drift rate (*v*), boundary separation (*a*), bias (*z*), and non-decision time (t) for the best-fit model (where drift rate varied by condition) are presented in Table 5. Figure 3 shows a visual representation of mean posterior differences by condition and stimulus type. Drift rates greater than zero reflect the rejection of words as self-descriptive, and drift rates less than zero represent the endorsement of words as self-descriptive. Therefore, for negative words, drift rates greater than zero represent the evidence accumulation rate for rejecting negative words as self-descriptive. For positive words, drift rates less than zero represent evidence accumulation for the endorsement of positive words as self-descriptive. Higher absolute drift rates equate to the faster speed of evidence accumulation. As depicted in Figure 3, there were no differences in drift rate for endorsing positive words in the blue-enriched condition (*v* = −0.71) when compared to the blue-depleted condition (*v* = −0.73; probability of overlap > .05). However, drift rates were significantly faster for rejecting negative words in the blue-enriched condition (*v* = 1.36) compared to the blue-depleted condition (*v* = 1.13; probability of overlap < .001). Absolute values of positive word drift rates were compared against negative word drift rates, which showed negative word drift rates were faster than positive word drift rates in blue-enriched and blue-depleted conditions (probability of overlap <.001 and <.001, respectively).

**Table 5.** Mean Regression Posterior Distributions of Drift Rate, Boundary Separation, and Non-decision Time in the Self-Referential Encoding Task (SRET)

	β	*SD*	95% CI
Positive word drift rate *(v)*			
Blue-depleted	−0.73	0.03	[−0.80, −0.67]
Blue-enriched	−0.71	0.04	[−0.78, −0.64]
Negative word drift rate *(v)*			
Blue-depleted	1.13	0.04	[1.06, 1.20]
Blue-enriched	1.36	0.04	[1.28, 1.44]
Boundary Separation (*a*)	1.42	0.05	[1.32, 1.52]
Bias (*z*)	0.49	0.01	[0.47, 0.52]
Non-decision time *(t)*	0.46	0.01	[0.44, 0.49]

β = mean of posterior distributions;

*SD,* standard deviation; CI, confidence interval.

**Figure 4.**
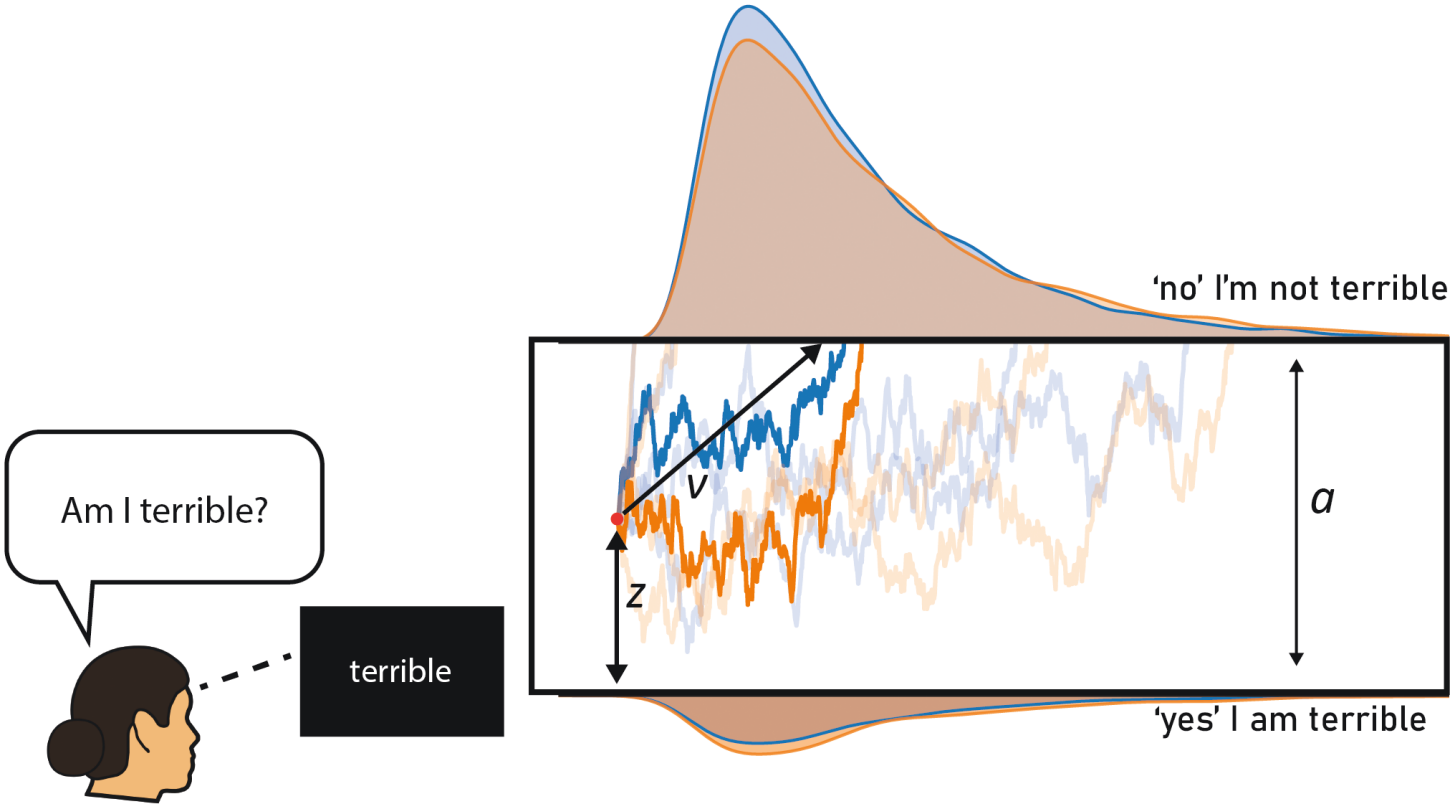
Simulated drift-diffusion decisions under blue-enriched and blue-depleted light conditions. The drift-diffusion figure shows the noisy simulated decision-making paths based on parameters estimated from the drift-diffusion model for the decision process. The paths for blue-enriched light are depicted in blue and in amber for blue-depleted light conditions. Under the blue-enriched light condition, there is a faster drift rate when rejecting negative words as self-descriptive, indicating greater ease in rejecting these descriptors as compared to the blue-depleted light condition.

In a comparison of samples estimated by the model compared to observed data for each light condition and stimulus type (depicted in Figure 3), we saw high coupling of observed RT and simulated quantiles for RT for each individual (calculated by estimated individual-level parameters and condition level parameter estimates of *v*), which suggests that the model was accurate at predicting observed data scores. A simulated decision based on group-level parameters is provided in Figure 4.

## Discussion

We investigated the effect of blue-enriched light on our perception of self, using the SRET. By contrasting blue-enriched and blue-depleted light conditions, we found a significant decrease in negative self-thoughts when exposed to blue-enriched light, indicated by increased odds of rejecting negative words as self-descriptive on an individual trial level. Through computational modeling of the drift-diffusion process, we found that exposure to blue-depleted light resulted in a slower accumulation of evidence (drift rate; *v*) when rejecting negative self-descriptors (e.g. terrible), indicating that deciding negative descriptors were unrelated to oneself was more difficult. The differential impact of light conditions was specific to negative words, with no differences observed in task responses or model parameters for positive self-descriptors. This pattern of results suggests that blue-enriched light facilitates the rejection of negative self-descriptors but does not facilitate the endorsement of a positive word. These results provide evidence for the direct effect of light on mood and cognition, and the positive effect of blue-enriched light in decreasing negative self-perceptions.

Using logistic mixed models, we analyzed trial-by-trial performance to assess the impact of light on negative self-referential processing. Compared to the trial-by-trial analysis, analyses of aggregated data (i.e. paired-sample t-tests of the proportion of response types) showed no significant differences between light conditions. It is likely that individual trial analysis preserved response variability within participants that was lost when data were averaged. Logistic-mixed effects models also found that longer RTs resulted in more negative self-evaluations for both positive and negative words: participants were less likely to reject negative words and less likely to endorse positive words as self-descriptive when taking more time to respond. Among participants, there was a tendency toward more negative self-assessment during prolonged response periods which may be due to greater decision uncertainty [25] and therefore a more difficult decision corresponding with longer RTs [16].

We observed no differences between conditions for the other drift-diffusion parameters: non-decision time (*t*), and bias (*z*), suggesting that basic perceptual and motor processes, as well as a priori response tendencies, were not affected by light conditions. For the group-level data, the bias parameter was close to 0.5 indicating no strong a priori bias towards “yes” or “no” responses in the task. We were unable to run models that allowed boundary separation (*a*) to vary due to failures of convergence, likely indicating that these models could not account for differences in RT distributions and responses between conditions. Furthermore, the RT distributions for negative words showed a pattern consistent with faster drift rates [16] for the blue-enriched condition, such as a leading edge and shifted tail for the blue-enriched condition when compared to the blue-depleted condition.

Although no previous studies have directly investigated the effect of blue-enriched light on drift-diffusion parameters and task responses in the SRET, differential SRET responses have been observed in disorders linked with low sensitivity of the circadian system to light [26] such as depression [12, 13]. Individuals who have experienced depression in the past have been found to exhibit slower drift rates in rejecting negative words as self-descriptive compared to healthy controls [12]. Furthermore, depressive symptom severity is correlated with both higher endorsement of negative words as self-descriptive and slower drift rates when rejecting such words [13]. Given that low sensitivity of the circadian system to light is hypothesized to increase the severity of depressive symptoms [26], one potential mechanism could be that lower light sensitivity induces negative self-perceptions and increases processing times for negative stimuli.

The SRET involves rumination due to the repetitive self-referential processing involved [27]. Previously, a connection has been demonstrated between rumination, negative self-referencing in individuals with depression, and increased amygdala reactivity [28]. Separately, light has been shown to suppress amygdala activity [29] and enhance connectivity between the amygdala and mood-regulating brain regions such as the ventromedial prefrontal cortex (vmPFC) [29] and the dorsolateral prefrontal cortex (dlPFC) [30]. Greater connectivity between the amygdala and prefrontal cortical areas has been hypothesized to enhance mood regulation through improved cognitive control [29, 30]. Specifically, amygdala-dlPFC communication is thought to enhance the down-regulation of emotional responses by attenuating the reactions of the amygdala [31]. Our findings are consistent with this amygdala reactivity hypothesis, as amygdala reactivity is observed in negative self-referencing, but not positive self-referencing [32]. Within the context of the SRET, this could facilitate an improved ability to reject negative words as self-descriptive, likely via downstream cognitive control of responses to negative adjectives.

Blue-depleted light may promote a more contemplative mental state, as slower drift rates could allow for the recruitment of processes that are not immediately available when decision-making begins. The integration of signal detection theory in conjunction with the DDM suggests that during periods of extended RTs (600 to 2000 ms), the direction of the noisy process (i.e. decision-making path) can ‘switch’ as decisions are influenced by multiple factors over time [33, 34]. For example, though a spider is not an insect, in early processing responses to the statement “a spider is an insect” might initially be perceived as *true*, but later in processing, with further recall of kingdom classification, this may be considered *false* (adapted from Ratcliffe et al., 2016). In our study, we observed that longer RTs were associated with decreased rejection of negative words, and decreased endorsement of positive words. This pattern might reflect greater “switching” of drift rate, leading to both an increased chance of reaching the opposing boundary (e.g. “yes, I am terrible,” and “no, I am not good”), and longer RTs. Furthermore, the slower drift rates observed in the blue-depleted condition may provide individuals with the extra time necessary to recruit recall processes and therefore incorporate self-relevant events in the decision-making process. This extended processing time could lead to introspective and critical self-thoughts.

Greater activity within the default mode network (DMN) during the blue-depleted light condition could explain both increases in observed negative self-referencing and potential enhanced recall of events [35]. The DMN has been linked to rumination, memory recall, and negative self-referencing [35, 36]. Additionally, the efficiency of the executive and cognitive control (task-positive) networks, which may operate counter to the DMN [37], is enhanced by blue light [38]. Non-visual photoreception pathways also project to DMN-associated regions such as the medial prefrontal cortex (mPFC) [10, 39], and downstream to the lateral habenula via the perihabenula [40, 41]. These convergent findings suggest that blue-depleted light conditions might reduce task-positive network activity and facilitate DMN activity, leading to negative self-referential thinking and possibly greater memory recall. However, DMN network activity is also related to creativity, in particular, divergent thinking (i.e. the generation of new ideas) [42]. There may be a trade-off between the mood-elevating effects of non-visual photoreception and DMN state creativity.

While our study provides insights into the effect of blue-enriched light on negative thoughts of self, future research can build on these in several ways. The menstrual phase was not captured during and between study visits, with menstrual cycle variations in mood regulation potentially introducing unmeasured variability across our 2-week interval between visits. However, we conducted models to assess whether the effect of light condition was moderated by sex, or explained by sex (i.e. as a main effect). Our analyses indicated that models including only RT and light conditions best explain the data for both positive and negative words, suggesting no significant sex-specific effects. Additionally, our current study did not directly examine the proposed influences of light on DMN connectivity and activity of the vmPFC, amygdala, and habenula. Future investigations could leverage functional magnetic resonance imaging to study the connectivity between frontal areas and the amygdala with non-visual photoreception and its influence on responses on the SRET to negative adjectives. We also suggest exploring whether amplified DMN activity enhances memory recall used in self-referential decisions, especially via parietal DMN regions [43]. Furthermore, employing electroencephalography (EEG) to measure event-related potentials in response to positive and negative SRET adjectives could clarify differential engagement with these adjectives [12, 44]. Furthermore, we suggest a novel paradigm of the SRET incorporating less extreme positively and negatively valanced self-descriptors. This paradigm could increase choice difficulty and result in more varied responses, enabling estimation of within-individual changes of boundary separation.

## Conclusion

Prior to the advent of modern electric lighting, our light environments were primarily dictated by the Earth’s rotation on its axis. Fires and incandescent lighting provided minimal blue wavelength content at night, thus minimally affecting the circadian system [45]. However, the transition to brighter, bluer, and more cost-effective LEDs has led to increased activation of non-visual photoreception [46, 47]. In contemporary societies, we observe ubiquitous engagement with artificial light: over-illumination of homes at night, use of large and bright LED screens for work and entertainment, and almost universal use of blue-enriched handheld devices [48]. Even if it is not consciously perceived, our findings show that blue light reduces negative thoughts, which may allow us to feel better about ourselves. With unlimited access to light in the modern era, we can resort to using light as a tool to manage feelings of low mood or sadness. Consequently, we may reach for light-emitting devices to abate melancholic feelings. Perhaps, unconsciously we are reinforcing a form of light-dependence, to avoid the *darkness*.

## Supplementary material

Supplementary material is available at *SLEEP* online.

zsaf034_suppl_Supplementary_Materials

## Data Availability

Data of this study are available from the corresponding author, SWC, upon reasonable request.
