# Supplementary material for: Blue light influences negative thoughts of self
Source: Sleep. 2025 Feb 25;48(7):zsaf034. doi: 10.1093/sleep/zsaf034 (PMC12246371; doi:10.1093/sleep/zsaf034)
Supplement: zsaf034_suppl_Supplementary_Materials [file zsaf034_suppl_supplementary_materials.docx]

**Blue Light Influences Negative Thoughts of Self.**

***Supplementary Document***

Malisa T. Burge; [malisa.burge@monash.edu](mailto:malisa.burge@monash.edu); School of Psychological Sciences and Turner Institute for Brain and Mental Health, Monash University, Melbourne, VIC, Australia

Ronel A. Lumapas; [ronel.lumapas02@gmail.com](mailto:ronel.lumapas02@gmail.com); School of Psychological Sciences and Turner Institute for Brain and Mental Health, Monash University, Melbourne, VIC, Australia

Alicia C. Lander; [alicia.lander@monash.edu](mailto:alicia.lander@monash.edu); Flinders Health and Medical Research Institute (Sleep Health), Flinders University, Bedford Park, SA 5042, Australia; School of Psychological Sciences and Turner Institute for Brain and Mental Health, Monash University, Melbourne, VIC, Australia

Brianna G. Thomas; [breethomas197@gmail.com](mailto:breethomas197@gmail.com); School of Psychological Sciences and Turner Institute for Brain and Mental Health, Monash University, Melbourne, VIC, Australia

Andrew J. K. Phillips; [ajk.phillips@flinders.edu.au](mailto:ajk.phillips@flinders.edu.au); Flinders Health and Medical Research Institute (Sleep Health), Flinders University, Bedford Park, SA 5042, Australia

Sean W. Cain; [Sean.Cain@flinders.edu.au](mailto:Sean.Cain@flinders.edu.au); Flinders Health and Medical Research Institute (Sleep Health), Flinders University, Bedford Park, SA 5042, Australia

**Corresponding author:** Sean Cain; [Sean.Cain@flinders.edu.au](mailto:Sean.Cain@flinders.edu.au); +61 467 387 031; Mark Oliphant Building, 5 Laffer Dr, Bedford Park, SA, 5042

**Data Analysis**

Logistic mixed effects models were completed in Rstudio R Version 4.2.2. using the package lme4 package [48]. An alpha level of .05 was used to determine significance. Dispersion was checked using the DHARMa package [22] (see Supplementary Figure 1 and 2). Logistic mixed effects models were compared using the BICcmodavg package to determine which model best fit the data [49] (see Supplementary Table 1 and 2). Data analysis was performed using the Hierarchical Drift Diffusion Model (HDDM) implemented on the MASSIVE High-Performance Computing (HPC) facility, owing to the computational expense of the models. Python 3.6.13 was utilised to operate HDDM version 0.8.0 [23]. Variants of a within-subjects regression (condition:stimulus) model with varying *v*, *t*, and *z*, and combinations of these parameters were run, with repeated effects of stimulus type, and light condition. For all models we specified no y-intercept so that we could look at each group parameter value. Additionally, for all models we specified the default method of outlier handling. All output traces for each model were saved to assess autocorrelation and convergence. Given the limited native support for Rhat calculations in HDDM regressor, four instances of the best-fit model with varied drift rate (v) were run. Trace values for each of these models were saved, and Rhat calculations were conducted using custom-written code to confirm model convergence (Supplementary Table 3).

**Order effects**

To investigate potential order effects of light conditions, we added an 'order' term to our best-fitting logistic mixed effects models. For negative words there was no effect of order (OR = 1.38, 95% CI [0.52, 3.68], *p* =.518). Additionally main effect of light condition (OR = 1.22, 95% CI [1.03, 1.44], *p* = .023) and the effect of reaction time remained similar (OR = 0.42, 95% CI [0.33, 0.53], *p* < .001) to the original best fitting model. For positive words, there was no effect of order (OR = 2.28, 95% CI [0.81, 6.45], *p* = .119). Additionally, the main effect of light condition (OR = 0.89, 95% CI [0.77, 1.03], *p* = 0.128) and effect of reaction time remained similar (OR = 0.32, 95% CI [0.25, 0.40], *p* < .001) to the original best fitting model.

**Supplementary Table 1. BICc-Based Comparison and Marginal Log-Likelihood (LL) of Logistic Mixed Effects Models Predicting Positive Self-Evaluation for Negative Words (Rejecting Negative Words)**

| Model ranking | Model equation | K | LL | BICc | delta BICc | BICc weight |
| --- | --- | --- | --- | --- | --- | --- |
| 1 | Positive self-evaluation ~ (1\|participant) + condition + rt | 4 | 3649.53 | 0 | 0.76 | 0.76 |
| 2 | Positive self-evaluation ~ (1\|participant) + condition + sex*rt | 6 | 3652.27 | 2.74 | 0.19 | 0.96 |
| 3 | Positive self-evaluation ~ (1\|participant) + condition*rt | 5 | 3656.79 | 7.27 | 0.02 | 0.98 |
| 4 | Positive self-evaluation ~ (1\|participant) + condition + sex + rt | 5 | 3657.92 | 8.39 | 0.01 | 0.99 |
| 5 | Positive self-evaluation ~ (1\|participant) + condition + hue+ rt | 5 | 3658.02 | 8.5 | 0.01 | 1 |
| 6 | Positive self-evaluation ~ (1\|participant) + condition*hue + rt | 6 | 3664.93 | 15.4 | 0 | 1 |
| 7 | Positive self-evaluation ~ (1\|participant) + condition*sex + rt | 6 | 3666.41 | 16.89 | 0 | 1 |
| 8 | Positive self-evaluation ~ (1\|participant) + condition + hue*rt | 6 | 3666.57 | 17.04 | 0 | 1 |
| 9 | Positive self-evaluation ~ (1\|participant) + condition*sex*rt | 9 | 3676.43 | 26.9 | 0 | 1 |
| 10 | Positive self-evaluation ~ (1\|participant) + condition*hue*rt | 9 | 3689.11 | 39.58 | 0 | 1 |
| 11 | Positive self-evaluation ~ (1\|participant) | 2 | 3693.52 | 43.99 | 0 | 1 |
| 12 | Positive self-evaluation ~ (1\|participant) + condition | 3 | 3695.33 | 45.8 | 0 | 1 |
| 13 | Positive self-evaluation ~ (1\|participant) + condition*sex | 5 | 3699.18 | 49.65 | 0 | 1 |
| 14 | Positive self-evaluation ~ (1\|participant) + condition + sex | 4 | 3703.74 | 54.21 | 0 | 1 |
| 15 | Positive self-evaluation ~ (1\|participant) + condition + hue | 4 | 3703.82 | 54.29 | 0 | 1 |
| *Note.* Condition = blue-enriched or blue-depleted light condition; rt = reaction time (s); sex = male or female participant; hue = awareness of differences in hues between sessions; delta BICc = difference in BIC score between the best model and respective model; BICc weight = proportion of the overall predictive power of contributed to by the respective model compared to the set of models; (1\|participant) = random intercept for each participant. Models are ordered for overall BICc weight. | | | | | | |

**Supplementary Table 2. BICc-Based Comparison and Marginal Log-Likelihood (LL) of Logistic Mixed Effects Models Predicting Positive Self-Evaluation for Positive Words (Endorsing Positive Words)**

| Model ranking | Model equation | K | LL | BICc | delta BICc | BICc weight |
| --- | --- | --- | --- | --- | --- | --- |
| 1 | Positive self-evaluation ~ (1\|participant) + condition + rt | 4 | 4425.37 | 0 | 0.94 | 0.94 |
| 2 | Positive self-evaluation ~ (1\|participant) + condition*rt | 5 | 4432.45 | 7.08 | 0.03 | 0.97 |
| 3 | Positive self-evaluation ~ (1\|participant) + condition + hue + rt | 5 | 4433.26 | 7.89 | 0.02 | 0.98 |
| 4 | Positive self-evaluation ~ (1\|participant) + condition + sex + rt | 5 | 4433.69 | 8.32 | 0.01 | 1 |
| 5 | Positive self-evaluation ~ (1\|participant) + condition*sex + rt | 6 | 4439.93 | 14.56 | 0 | 1 |
| 6 | Positive self-evaluation ~ (1\|participant) + condition + sex*rt | 6 | 4440.25 | 14.88 | 0 | 1 |
| 7 | Positive self-evaluation ~ (1\|participant) + condition + hue*rt | 6 | 4441.21 | 15.84 | 0 | 1 |
| 8 | Positive self-evaluation ~ (1\|participant) + condition*hue + rt | 6 | 4441.28 | 15.91 | 0 | 1 |
| 9 | Positive self-evaluation ~ (1\|participant) + condition*hue*rt | 9 | 4460.88 | 35.51 | 0 | 1 |
| 10 | Positive self-evaluation ~ (1\|participant) + condition*sex*rt | 9 | 4461.73 | 36.36 | 0 | 1 |
| 11 | Positive self-evaluation ~ (1\|participant) | 2 | 4510.49 | 85.12 | 0 | 1 |
| 12 | Positive self-evaluation ~ (1\|participant) + condition | 3 | 4518.28 | 92.91 | 0 | 1 |
| 13 | Positive self-evaluation ~ (1\|participant) + condition + hue | 4 | 4525.85 | 100.48 | 0 | 1 |
| 14 | Positive self-evaluation ~ (1\|participant) + condition + sex | 4 | 4526.64 | 101.27 | 0 | 1 |
| 15 | Positive self-evaluation ~ (1\|participant) + condition*sex | 5 | 4533.59 | 108.22 | 0 | 1 |
| *Note.* Condition = blue-enriched or blue-depleted light condition; rt = reaction time (s); sex = male or female participant; hue = awareness of differences in hues between sessions; delta BICc = difference in BIC score between the best model and respective model; BICc weight = proportion of the overall predictive power of contributed to by the respective model compared to the set of models; (1\|participant) = random intercept for each participant. Models are ordered for overall BICc weight. | | | | | | |

| **Supplementary Table 3. Trace values of best fit model (where only drift rate *v* varied) confirming convergence across four different models.** | |
| --- | --- |
| a | 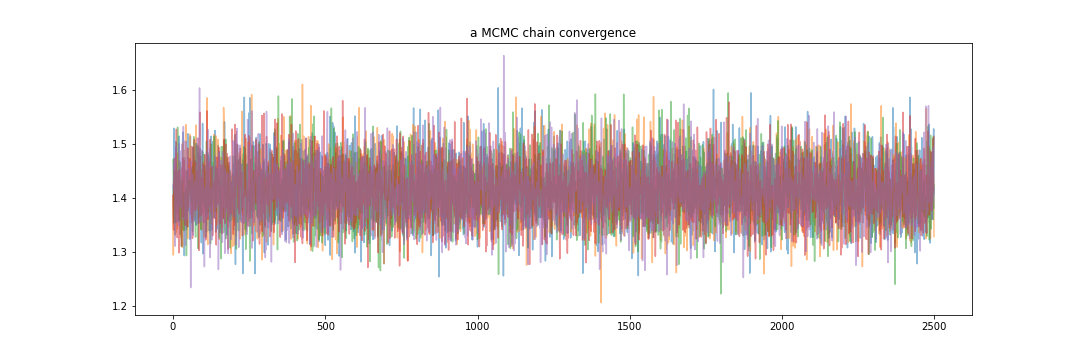 |
| a_std | 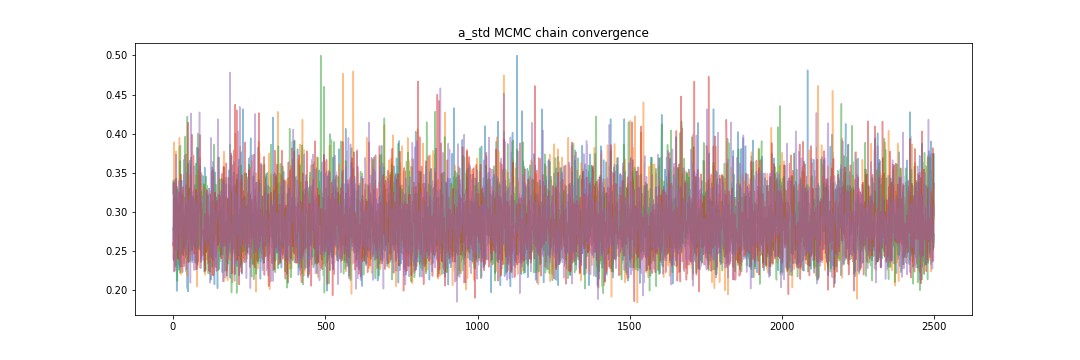 |
| t | 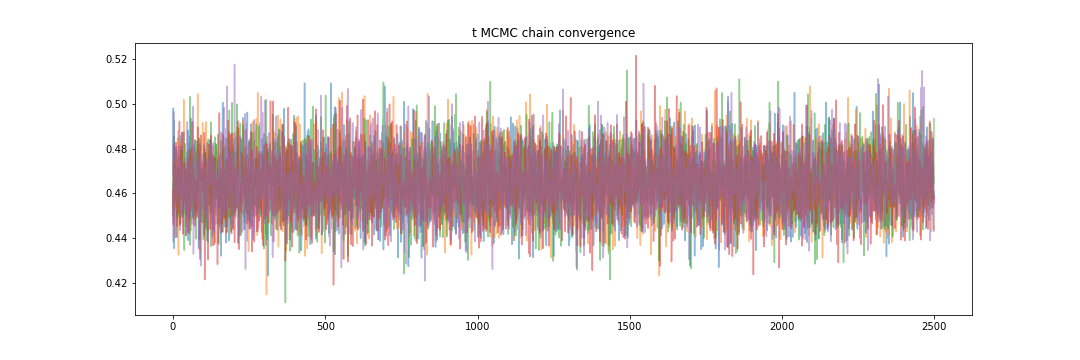 |
| t_std | 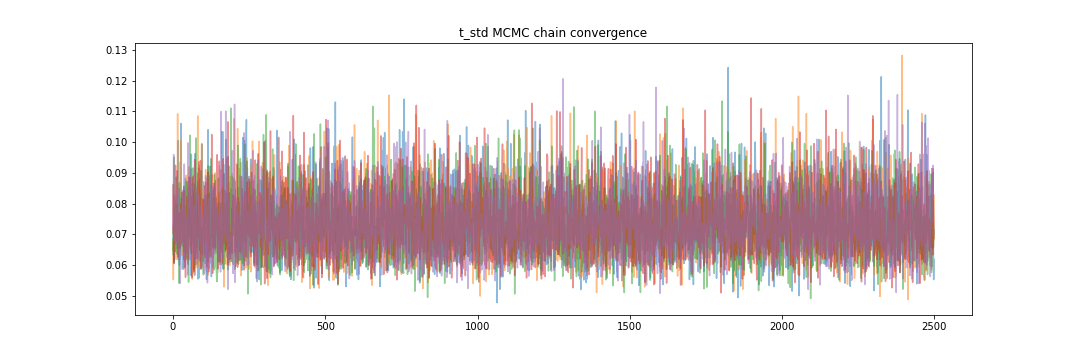 |
| v0_neg | 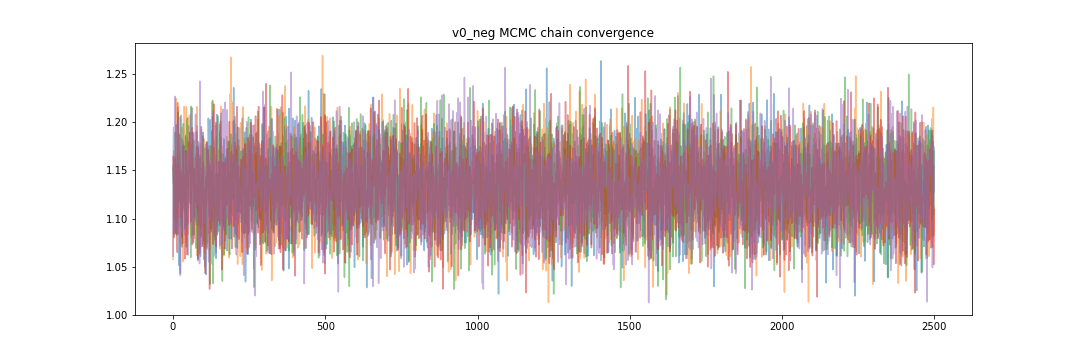 |
| v0_pos | 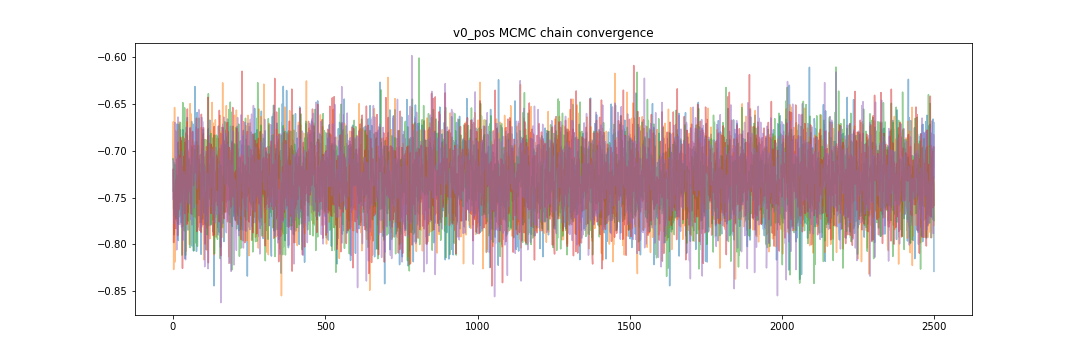 |
| v1_neg | 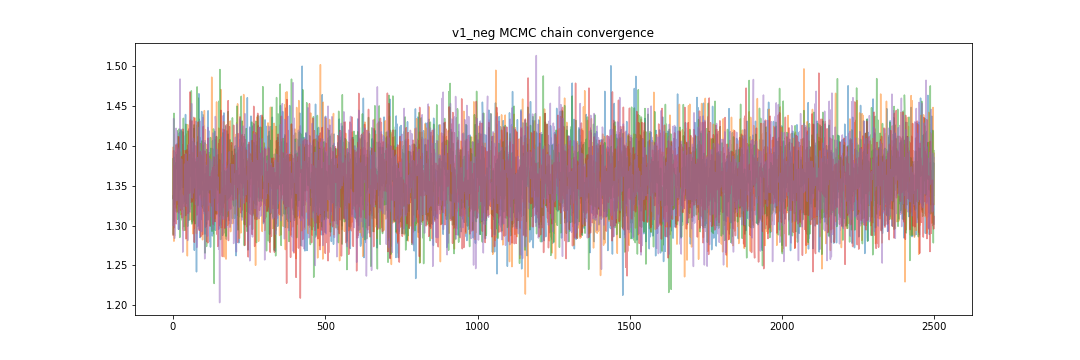 |
| v1_pos | 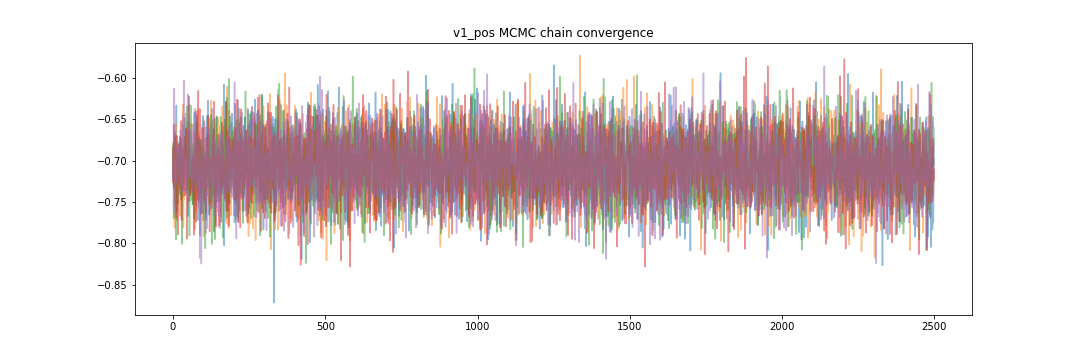 |
| z | 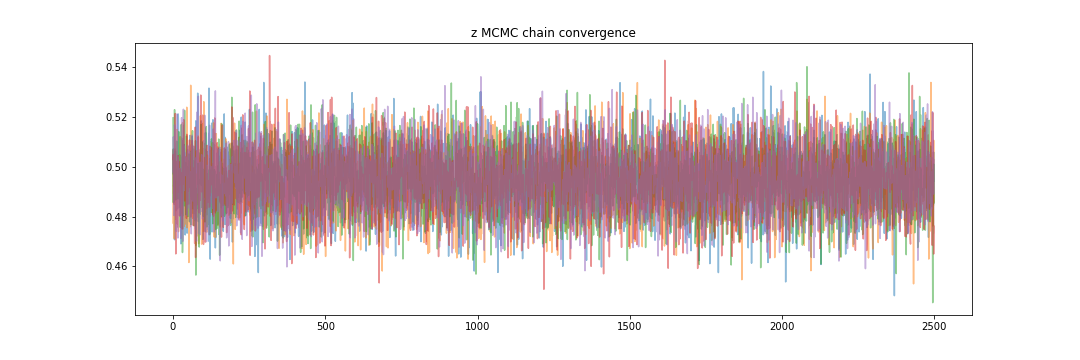 |
| z_std | 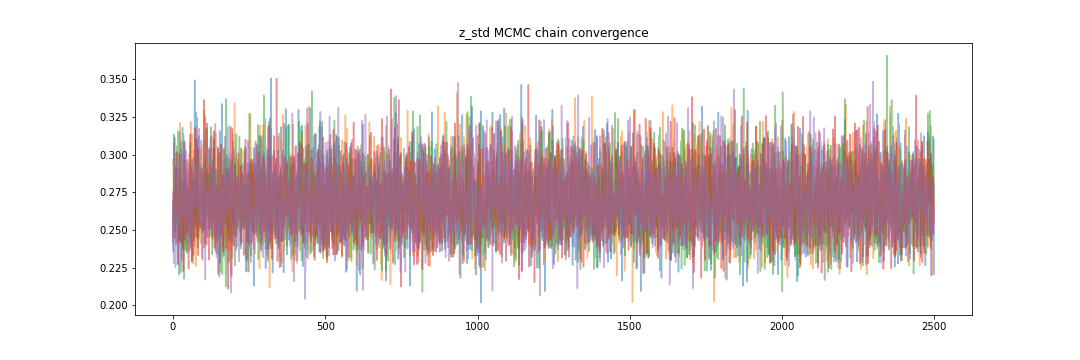 |

**Supplementary Figure 1.**


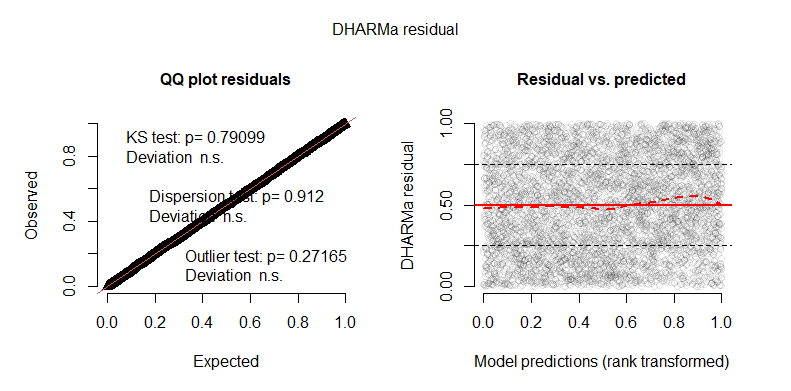


Supplementary Figure 1. Dharma plot for positive words for best fit model, formula: Positive self-evaluation ~ (1|participant) + condition + reaction time. Plot indicating normality distributed residuals, no significant residual dispersion, and no significant residual outliers.

**Supplementary Figure 2.**


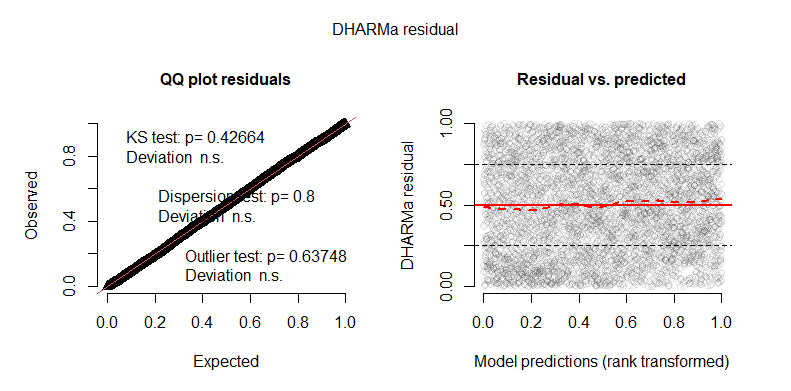


Supplementary Figure 2. Dharma plot for negative words for best fit model, formula: Positive self-evaluation ~ (1|participant) + condition + reaction time. Plot indicating normality distributed residuals, no significant residual dispersion, and no significant residual outliers.
